# Medico-legal assessment of personal damage in older people: report from a multidisciplinary consensus conference

**DOI:** 10.1007/s00414-020-02368-z

**Published:** 2020-07-17

**Authors:** Francesca Ingravallo, Ilaria Cerquetti, Luca Vignatelli, Sandra Albertini, Matteo Bolcato, Maria Camerlingo, Graziamaria Corbi, Domenico De Leo, Andrea De Nicolò, Francesco De Stefano, Alessandro Dell’Erba, Paola Di Giulio, Ranieri Domenici, Piergiorgio Fedeli, Alessandro Feola, Nicola Ferrara, Paola Forti, Francesca Frigiolini, Pasquale Gianniti, Enrico Gili, Primiano Iannone, Alessandro Lovato, Maria Lia Lunardelli, Alessandra Marengoni, Franco Marozzi, Massimo Martelloni, Patrizia Mecocci, Andrea Molinelli, Lorenzo Polo, Margherita Portas, Patrizio Rossi, Carlo Scorretti, Marco Trabucchi, Stefano Volpato, Riccardo Zoja, Gloria Luigia Castellani

**Affiliations:** 1 Ageing Research Centre, Department of Medical and Surgical Sciences (DIMEC), University of Bologna, Via Irnerio 49, 40126 Bologna, Italy; 2 Department of Legal Medicine, ASUR Marche AV4, Fermo, Italy; 3 IRCCS Istituto delle Scienze Neurologiche di Bologna, Bologna, Italy; 4 Senior Italia, Bologna, Italy; 5 National Board of Young Medico-legal Experts, Legal Medicine, University of Padua, Padua, Italy; 6 Bologna, Italy; 7 Department of Medicine and Health Sciences “V. Tiberio” and Italian Society of Gerontology and Geriatrics, University of Molise, Campobasso, Italy; 8 College of Italian Professors of Legal Medicine, Department of Diagnostics and Public Health, Unit of Forensic Medicine, University of Verona, Verona, Italy; 9 Turin, Italy; 10 Italian Society of Legal and Insurance Medicine (SIMLA), Department of Science of Health (DISSAL), University of Genoa, Genoa, Italy; 11 Federation of the Italian Associations of Medico-Legal Experts (FAMLI), Department of Interdisciplinary Medicine (DIM), Section of Legal and Forensic Medicine, University of Bari, Bari, Italy; 12 Department of Public Health and Paediatrics, University of Turin, Turin, Italy; 13 Pisa, Italy; 14 Italian Research Group on Personal Injury (GISDAP), University of Camerino, Camerino, Italy; 15 National Board of Young Medico-legal Experts, Department of Experimental Medicine, University of Campania “Luigi Vanvitelli”, Naples, Italy; 16 Department of Translational Medical Sciences and Italian Society of Gerontology and Geriatrics, Federico II University of Naples, Naples, Italy; 17 Department of Science of Health (DISSAL), University of Genoa, Genoa, Italy; 18 Supreme Court of Cassation, Rome, Italy; 19 National Association of Insurance Companies (ANIA), Rome, Italy; 20 National Center for Clinical Excellence, Quality and Safety of Care (CNEC), Rome, Italy; 21 The Surveillance and Cooperation Body on Civil Justice, Bologna, Italy; 22 Geriatric Unit, Orthogeriatric Ward, University Hospital Policlinico S. Orsola Malpighi, Bologna, Italy; 23 Department of Clinical and Experimental Sciences, University of Brescia, Brescia, Italy; 24 Federation of the Italian Associations of Medico-Legal Experts (FAMLI), Milan, Italy; 25 Department of Legal Medicine, Local Health Trust Toscana Nordovest, Scientific Society of Forensic Medicine of Italian National Health Service Hospitals (COMLAS), Lucca, Italy; 26 Department of Medicine, Institute of Gerontology and Geriatrics, University of Perugia, Perugia, Italy; 27 Pavia, Italy; 28 Verona, Italy; 29 National Institute for Insurance against Accidents at Work (INAIL), Rome, Italy; 30 Trieste, Italy; 31 Italian Association of Psychogeriatrics (AIP), University of Tor Vergata, Rome, Italy; 32 Department of Medical Sciences, University of Ferrara, Ferrara, Italy; 33 Department of Health and Biomedical Sciences, Section of Legal Medicine, University of Milan, Milan, Italy; 34 Federation of the Italian Associations of Medico-Legal Experts (FAMLI), Verona, Italy

**Keywords:** Medico-legal assessment, Personal damage, Personal injury, Older adults, Pre-existing conditions, Multidimensional assessment

## Abstract

Ageing of the global population represents a challenge for national healthcare systems and healthcare professionals, including medico-legal experts, who assess personal damage in an increasing number of older people. Personal damage evaluation in older people is complex, and the scarcity of evidence is hindering the development of formal guidelines on the subject. The main objectives of the first multidisciplinary Consensus Conference on Medico-Legal Assessment of Personal Damage in Older People were to increase knowledge on the subject and establish standard procedures in this field. The conference, organized according to the guidelines issued by the Italian National Institute of Health (ISS), was held in Bologna (Italy) on June 8, 2019 with the support of national scientific societies, professional organizations, and stakeholders. The Scientific Technical Committee prepared 16 questions on 4 thematic areas: (1) differences in injury outcomes in older people compared to younger people and their relevance in personal damage assessment; (2) pre-existing status reconstruction and evaluation; (3) medico-legal examination procedures; (4) multidimensional assessment and scales. The Scientific Secretariat reviewed relevant literature and documents, rated their quality, and summarized evidence. During conference plenary public sessions, 4 pairs of experts reported on each thematic area. After the last session, a multidisciplinary Jury Panel (15 members) drafted the consensus statements. The present report describes Conference methods and results, including a summary of evidence supporting each statement, and areas requiring further investigation. The methodological recommendations issued during the Conference may be useful in several contexts of damage assessment, or to other medico-legal evaluation fields.

## Introduction

The number of people aged 65 years and over (older people) is increasing worldwide, particularly in North America and Europe [1]. Among the European Union member states, Italy has the highest number of older people in the total population (22.3%), followed by Greece (21.5%), and Germany (21.2%) [2]. These numbers are bound to increase in the coming years. For instance, the percentage of older people is expected to rise to 34% of the total population by 2045–2050 in Italy [3]. Increasing longevity and active ageing are associated with a higher occurrence of traumatic injuries in elderly people. In Italy, the number of road accident injuries in people over 60 has almost doubled in the past 30 years (21,379 injuries in 1987 vs. 42,320 injuries in 2017) [4]. People aged over 60 represent 36.5% of total male and 60.4% of total female patients accessing Emergency Department (ED) care for accidental injuries, and 41.6% of all patients accessing ED care for household accidents [5].

This scenario represents a challenge for national healthcare services and for healthcare professionals, including Medico-Legal experts. Indeed, the latter assess the personal damages resulting from traumatic injuries in an increasing number of older people, especially in the legal framework of third-party motor liability [6].

The ascertainment and evaluation of personal damage are complex issues from both clinical and medico-legal standpoints [7]. The assessment of personal damage in older people can be an extremely complex process because of the functional and physio-pathological modifications due to ageing, the heterogeneity of the older population with regard to functional status, the scarcity of validated tools for the functional assessment of older people in the medico-legal context, and the requirement to adjust the medico-legal examination procedures, timing, and duration to the needs of the ageing population. For this reason, the guide to the medico-legal evaluation of personal damage recently issued by the Italian Society of Legal and Insurance Medicine (SIMLA) contains a chapter on the “Evaluation methods in older people” [8]. In addition, the most used compensation tables and barèmes (rating tables) in the medico-legal practice only take into account the consequences of a specific injury without considering the age and the pre-existing conditions of the injured person. How to include these aspects in the rating of personal damage in older people, who frequently lack complete psychophysical integrity, is still a matter of debate. Finally, there is no standard procedure to assess the consequences of minor injuries that might severely impair older people (e.g., a foot fracture that impairs the autonomous mobility of an older person), even because for actuarial reasons damage compensation is progressively reduced with the age.

The impact of the abovementioned assessment issues on the daily practice of medico-legal experts was confirmed by interviews with experts in personal damage assessment carried out in December 2017, and represents the starting point of the Consensus Conference on Medico-Legal Assessment of Personal Damage in Older People.

The aim of a consensus conference is to produce recommendations through “the analysis of the available evidence on specific controversial subjects for which there is no shared opinion, and often leading to non-standard practices in the clinical, organisational, and managemental aspects of healthcare” [9]. In fields where the scarcity of evidence hinders the development of formal guidelines, consensus statements are a useful resource in supporting healthcare providers’ decision-making [10].

## Objectives

The Consensus Conference on Medico-Legal Assessment of Personal Damage in Older People focuses on the medico-legal assessment of personal damage (both as unbiased ascertainment and rating) in the elderly person. The consensus conference aims to:

- Improve knowledge on the subject
- Define standard medico-legal assessment procedures
- Agree upon a common terminology
- Identify qualitative indices, and
- Suggest areas requiring further investigation

The consensus conference target audience includes Legal Medicine experts and Geriatricians, and insurance and law professionals in the field of personal damage, as well as the lay public. Also, the consensus conference’s recommendations can help all physicians (General Practitioners in the first place) who take care of older people.

The consensus conference was held in Bologna (Italy) on June 8, 2019. This report describes the Conference methods, presents the summary of evidence, and the statements/recommendations formulated by the Jury Panel, which also pointed out limitations and areas requiring further investigations.

## Methodology

The consensus conference, promoted and endorsed by several Italian Scientific Societies and Institutions (see Appendix 1), was organized according to the U.S. National Institute of Health Consensus Development Program standards by following the guidelines issued by the Italian National Institute of Health (ISS, Rome, Italy) [9]. The methodology was adapted to hold the Conference in a single day [11].

### Consensus conference organization

The organization of the consensus conference was a 2-year long process (May 2017–May 2019), preceded by a 5-month evaluation period during which the feasibility of the project was assessed (December 2016–April 2017). The early phases of conference organization involved the constitution of (1) the Promotion Committee, which promoted the conference, appointed the Scientific Technical Committee (STC) members, identified the field experts, and solicited questions concerning selected topics; (2) the STC, which selected the members of the Scientific Secretariat, prepared the questions, and drafted the guidelines for the literature review and the preparation of conference presentations; and (3) the Scientific Secretariat (including a literature review expert) was in charge of reviewing the scientific literature and preparing summary documents containing the information collected during the review.

Consensus Conference Promoters identified 8 field experts (4 in Legal Medicine and 4 in Geriatrics), responsible for providing the Jury Panel with the summary of evidence and presenting it during the Conference. To constitute a multidisciplinary and multiprofessional Jury Panel, multiple stakeholders (i.e., Scientific Societies and Institutions, National Authorities, medical, legal, and insurance organizations) were asked to designate a representative who would sit on the Jury Panel. The Jury Panel also included a nursing professor with an interest in geriatric research, a methodologist specialized in searching for and assessing evidence, and a representative of elderly people’s associations. The Conference organogram is presented in Appendix 1. All participants signed a Declaration of Interest and a Confidentiality Agreement.

### Conference questions and literature review

The 16 consensus conference questions addressed the following 4 thematic areas:

1. Evidence that traumatic injury outcomes are different between older and younger people and its relevance for the assessment of personal damage (general part)
2. Pre-existing status reconstruction and evaluation
3. Medico-legal examination procedures
4. Multidimensional assessment and scales

All questions focused on personal damage assessment in older people. Starting from questions, a systematic literature review for each thematic area/topic of the conference was carried out. To deal with the great variability of possible clinical conditions, the necessity of also including theoretical/doctrinal documents, the existence of a considerable amount of literature, and the difficulty of identifying documents inherent to the medico-legal practice, the systematic review followed some general criteria. First, a hierarchical search strategy favouring the selection of the most recently published systematic reviews was applied. Non-systematic reviews were included when systematic reviews were not available. Primary studies were included if no reviews were available on a specific topic, or if primary studies were more recent than systematic reviews on the same topic. Then, for questions concerning clinical practices and medico-legal technical aspects, the search focused on available guidelines, narrative review, and grey literature.

In particular, for the first thematic area, only reviews discussing trauma outcomes in general or major trauma outcomes were included, excluding those that did not analyze associations between outcomes and age ≥ 65 years. For questions on pre-existing status, only reviews investigating the role of the pre-injury status on trauma outcomes in older people and theoretical/doctrinal documents were included. For questions concerning examination procedures, when reviews were not available, observational studies were included. For questions concerning the multidimensional assessment, guidelines, evidence-based synthesis, health technology documents, documents by scientific societies, and primary studies on the functional status assessment tools validated in Italian were included. Also, experts were asked to recommend additional documents, which were included only if they matched the above-mentioned criteria.

A first round of literature search was performed between July and August 2018, using the PubMed and EMBASE databases (including MEDLINE in-process, other non-indexed citations); the search led to the identification of 3924 documents, most of which were of little or no relevance to the Conference questions. Therefore, at the beginning of 2019, a second round of literature search, that included documents published until the end of 2018, was performed. The new search led to the identification of 5437 articles, 29 of which were included in the review. Theoretical/doctrinal documents and court rulings were identified from the list of non-indexed journals of Legal Medicine that publish articles on the topic, and the DeJure (https://dejure.it) database. This search led to a list of 10 relevant documents. The CISMeF (www.chu-rouen.fr/cismef), Cochrane Library (www.cochranelibrary.com), and NHS Evidence (www.evidence.nhs.uk) databases were also used for the literature search. For grey literature retrieval, webpages of scientific societies, point-of-care evidence-based medicine websites (UpToDate), as well as Google and Google Scholar search engines were used. This search produced 10 relevant documents, to which 1 document recommended by an expert was added.

Four couples of reviewers performed a blind inclusion procedure and literature assessment, supported by the literature review expert and the STC members. In summary, 2 guidelines [12, 13]; 8 evidence-based synthesis, health technology documents, and documents by scientific societies [14–21]—in the following, we will indicate with “synthesis of evidence documents” these documents—7 systematic reviews [22–28]; 3 non-systematic reviews [29–31]; 20 primary studies [32–51]; and 10 theoretical/doctrinal documents [7, 8, 52–59] were included in the review. Thirty three out of the 50 documents were in English, 16 in Italian, and 1 in French; only 45 out of the 50 selected documents were used by the Jury Panel to formulate the consensus statements.

For each thematic area/topic, the Scientific Secretariat prepared a table summarizing the evidence, reporting details of each publication, including author/s of the study, year of publication, publication characteristics, type of document, condition under investigation, number of patients included in the study and their characteristics, total number of studies included in the review (when applicable), main measured outcomes, follow-up duration, and main findings with reference to the questions. Similarly, the Scientific Secretariat prepared summary tables for the theoretical/doctrinal material and other included documents. The experts and Jury Panel members were provided with the reviewed documents, together with the summary tables of the results of the quality assessment (see below), ahead of consensus conference. The experts used a further 20 non-reviewed documents during their presentations of summary of evidence.

### Assessment of the quality of evidence

The following tools were used for the evaluation of documents included in the revision: *AGREE II* (Italian version) [60], for guidelines; *AMSTAR 2* [61] for systematic reviews, meta-analyses, and non-systematic reviews; the *Standard Quality Assessment Criteria for Evaluating Primary Research Papers from a Variety of Fields* [62] for primary studies. Two reviewers independently evaluated each document. To homogeneously classify the quality level of the documents, quality indices were assigned according to a quartile grading scale (Table 1).

**Table 1:** Assessment of literature quality

Authors, year (reference number)	Document type	Quality rating^a^
Abete et al. 2017 [32]	Primary study	****
Britt et al. 2005 [33]	Primary study	***
Brown et al. 2017 [22]	Systematic review	***
Callahan et al. 2000 [34]	Primary study	***
Callahan et al. 2004 [35]	Primary study	****
Chipi et al. 2018 [36]	Primary study	****
Clayman et al. 2005 [37]	Primary study	****
Corbi et al. 2018 [38]	Primary study	****
Deveugele et al. 2002 [39]	Primary study	****
Ferrari et al. 2017 [40]	Primary study	****
Gardner et al. 2018 [29]	Narrative review	*
Girtler et al. 2012 [41]	Primary study	***
Hashmi et al. 2014 [23]	Systematic review	*
Hildebrand et al. 2016 [30]	Review	*
Hogan et al. 2011 [31]	Review	*
Ishikawa et al. 2005 [42]	Primary study	***
Laidsaar-Powell et al. 2013 [24]	Systematic review	**
McIntyre et al. 2013 [25]	Systematic review	*
New Zealand Guidelines Group 2003 [12]	Guideline	****
Petek Ster et al. 2008 [43]	Primary study	****
Regione Toscana 2017 [13]	Guideline	***
Reuben et al. 2004 [44]	Primary study	****
Sammy et al. 2016 [26]	Systematic review	**
Sawa et al. 2018 [27]	Systematic review	***
Schmidt et al. 2009 [45]	Primary study	***
Storti 2009 [46]	Primary study	***
Tähepold et al. 2003 [47]	Primary study	***
Wolff et al. 2008 [48]	Primary study	****
Wolff et al. 2011 [28]	Systematic review	**
Wolff et al. 2012 [49]	Primary study	****
Wolff et al. 2017 [50]	Primary study	**
Wooldridge et al. 2010 [51]	Primary study	****

^a^Overall quality quartiles of guidelines: * 0–24, ** 25–49, *** 50–74, **** 75–100; Overall confidence rating of the results of reviews, systematic reviews and meta-analyses: * critically low, ** low, *** average, **** high; Overall quality quartiles of primary studies: * summary score < 25%, ** summary score 25–49%, *** summary score 50–74%, **** summary score 75–100%

Overall, the quality of the observational studies was good, while that of systematic and non-systematic reviews was poor (there were no high quality systematic reviews); one of the guidelines was of high quality, while the other was of average quality (Table 1). Theoretical/doctrinal documents and synthesis of evidence documents were excluded from the quality assessment.

### Consensus conference day and following steps

The consensus conference was held on June 8, 2019, in Bologna (Italy). The invited audience included field experts and citizens.

During the morning sessions, the experts presented a summary of the literature before the Jury Panel and the audience. For each session, the speakers were a medico-legal expert and a geriatrician giving a 40-min-long oral presentation, followed by 30 min of discussion. After the last session, the Jury Panel gathered in a secluded place to discuss the evidence presented and to draft a preliminary consensus document including the answer to each question (i.e., the Jury Panel statements/recommendations) that was publicly presented by the Jury Panel’s Chair at the end of the Conference.

The final version of the consensus document was drafted during the months following the conference, and finally approved on February 29, 2020. The agreement on the Jury Panel statements was reached through a two-round Delphi-like method [11]. According to the consensus conference guidelines [9], during this stage the Jury Panel members could add only minor modifications and revisions to the preliminary statements/recommendations.

## Summary of evidence

### Evidence that traumatic injury outcomes are different between older and younger people and its relevance for the assessment of personal damage

Two reviews suggest that after traumatic injuries older people, regardless of injury type, have a lower quality of life, longer recovery times, less probability to regain independence in activities of daily living, and a reduced functional status [22, 30]. Good pre-injury functions (especially motor function), and pre-injury independent living can predict a better outcome [22].

Several reviews found a direct association between ageing and mortality after major traumas [23, 25–27, 30]. However, it is not clear whether age represents an independent factor, and it is generally difficult to establish an age threshold over which mortality significantly increases [29, 30]. Additional factors that should be considered are the severity of the injury [23, 27], and the gender (elderly men have higher risks of mortality compared to women) [26]. The role of pre-existing comorbidities and/or pharmacological treatments is still controversial [29, 30], although some studies suggest that polypharmacy represents a crucial factor in making older people more vulnerable to mortality after even a slight exposure to modest stress [23].

In conclusion, several studies show that the elderly population experience worse outcomes with regard to mortality, complications, functional status, and quality of life compared to a younger population. Different factors, including the pre-existing functional status, contribute to an increased risk of post-traumatic unfavourable outcomes and mortality in older people. However, further studies are required to better understand the role of these factors and their interactions with age.

According to experts’ opinions, available evidence is relevant to the evaluation of personal damage with regard to the *an* (general and individual causation) and the *quantum* (methods for damage ascertainment and rating criteria). Concerning the causation, these lines of evidence provide an empirical unbiased basis to establish that, on average, injury outcomes are more severe in a specific group of older people compared to the reference group composed of younger people (general causation). In addition, these lines of evidence provide objective support in explaining the particular severity of the outcomes in that specific older person undergoing a medico-legal assessment (individual causation). In this assessment, the evidence of the harmful mechanism has a key role, and should always be assessed in the ascertainment of causation [63]. The understanding of causal mechanisms should also be fundamental in the context of *evidence*-*based medicine* [64]. With regard to the *quantum*, the criteria used in current rating methods depend upon decisions not stemming from empirical evidence, but available evidence might support these decisions in a concrete manner.

Questions and Jury Panel statements/recommendations are reported in Table 2.

**Table 2:** General part: questions and Jury Panel statements/recommendations

Level of evidence	Questions and Jury Panel statements/recommendations
Systematic reviews (2 of average quality, 1 of low quality, 2 of very low quality), 2 non-systematic reviews of very low quality, and the experts’ opinions	1.1 Is there evidence that traumatic injury outcomes are different between older and younger people? *There is evidence that the most unfavourable outcomes of a traumatic injury are usually associated with ageing. However*, *different old age-related factors may contribute to the risk of worse outcomes*. 1.2 Which medico-legal relevance does this evidence have for personal damage assessment? *A comprehensive and personalized assessment of age-related critical factors is essential for the evaluation of personal damage in the medico*-*legal context.*

### Reconstruction and evaluation of the pre-injury health status

The available literature on this topic consists mainly of theoretical/doctrinal contributions.

#### Reconstruction of the pre-injury status

According to the doctrine, the reconstruction of the pre-existing status of the injured party is essential [7, 52, 56, 65]. The pre-existing status implies “the entirety of the physiological and pathological conditions existing before the medico-legal-related event happened” [66]. In general, evaluating the injury without considering the effects it may exert on the general homeostasis (stability) of the elderly person is considered inaccurate [8]. Indeed, in an older person, even a mild injury may cause more severe physical and psychological consequences compared to a younger person [8, 53, 58], in line with the “locality principle” (i.e., an injury in a specific area of the body might impair the functionality of the whole person) [59]. A multidimensional assessment of the global health status helps predict the post-operative and post-therapy outcomes (surgery and therapy being considered as traumatic events) [67, 68], regardless of the affected body area.

In order to reconstruct the pre-existing status, several studies recommend the need to investigate, besides pre-existing diseases and therapies, the following aspects: what the person was able to do beforehand [57]; clinical factors (inactive lifestyle, nutritional conditions, obesity, stress, alcohol and tobacco use, weight, memory impairments); autonomy in activities of daily living; social disadvantage [52]; education, work experiences, malformations, and consequences of previous injuries/diseases [56]; and frailty [8]. Frailty and physical performance (i.e., mobility and level of independence/autonomy) are generally considered the best indicators of the health status in the elderly person [69].

Some authors believe that the reconstruction of pre-existing status should only rely on objective information: ongoing treatments, General Practitioner’s opinion, laboratory and diagnostic tests, family context, and personal autonomy (i.e., independent living, care requirements, hobbies) [52]. Others recommend considering information obtained from the patient and the patient’s family, along with welfare-related documentation (i.e., civil incapacity, social security provisions) [8]. The experts acknowledge the importance of pursuing the collaboration of elderly patients if possible, since it represents a key factor for the clinical medico-legal judgement.

#### Medico-legal evaluation of pre-existing status modification

According to some authors, the evaluation should not only rely on standard ratings but should consider the actual negative impact of the injury on the general homeostatic balance of the person [54], including overall modifications of the party’s pre-existing status [8]. Elderly people should not be considered as people with disabilities and/or impairments [70] if their general conditions, albeit physiologically reduced in various domains compared to younger people, are in line with the age standards. During personal damage assessment, standard conditions for older people correspond to the maximum integrity value (100%) and the damage assessment procedure should refer to this value, and determine the relative reduction of the psychophysical integrity following the injurious event [55].

Questions and Jury Panel statements/recommendations are reported in Table 3.

**Table 3:** Pre-existing status reconstruction and evaluation: questions and Jury Panel statements/recommendations

Level of evidence	Questions and Jury Panel statements/recommendations
Theoretical/doctrinal contributions, and the experts’ opinions	2.1 Under which circumstances should a medico-legal expert, besides assessing the pre-injury status of the affected area, also examine the general pre-injury status of an older person? *The reconstruction of the pre-existing status represents a fundamental step in any medico*-*legal assessment process*.
	2.2 What information about the pre-existing status should always be collected? *Data concerning the pre-existing status should include information on pre-existing diseases, pharmacological therapies*, *ageing process*, *physical and cognitive performance*, *and social relationships*. *The evaluation of the pre-existing status should be aimed at defining what the person was able to do beforehand*, i.e., *sedentary lifestyle*, *and autonomy in feeding*, *ambulating*, *dressing*, *personal hygiene and in body functions*; *social skills and relationship level.*
	2.3 Which methodology should be followed to achieve a reliable reconstruction of the pre-existing status? *The optimal procedure to obtain a valid reconstruction of pre-injury conditions relies mainly on:* *a*) *Anamnesis* (*clinical history*) *obtained from the patient, family and/or caregivers, and clinical documents provided;* *b*) *Legally obtainable medical data and information, including access to formal and informal care resources* (*day care centres*, *supplementary home care*, *formal caregivers*). *Information collection must comply with privacy and personal data protection laws and should not be cause of action estoppel/trial issue preclusion*/*collateral estoppel*. 2.4 How should the modification of the pre-existing status in older people be evaluated from a medico-legal standpoint? *An evaluation that considers the physical and cognitive performance of the aged person before the injury is highly recommended*. *The evaluation criteria should consider the actual reduction of the patient*’*s pre*-*existing psychophysical status and should not be necessarily limited to the barème indications that are developed with reference to the impairment due to single injuries affecting a theoretical integrity*.

### Examination procedures for the medico-legal ascertainment of personal damage in older people

#### Criteria to establish the minimum time interval before the injured person undergoes medico-legal examination

A search of the literature did not provide results on the topic, partly because an unambiguous definition of clinical stability is still lacking [71]. Some studies suggest that multidimensional assessment scales may help define the stabilization parameters of specific injuries (e.g., femur fractures) and acute conditions [72–75]. However, there is no conclusive evidence in this regard.

#### Duration of the medico-legal examination in an older person

Several observational studies found that the time needed for a medical examination increases with ageing and in the case of elderly patients [33, 34, 39, 43, 47, 51]. Only one study did not find specific associations [35]. In general, a longer examination duration was related to the greater complexity of the elderly patient, due to comorbidity and polytherapy (and their impact on clinical history collection), and specific psychological and social conditions that characterize this age range.

#### Participation of a family member and/or caregiver in the medical-legal visit

Several observational studies showed that accompanying family members and/or caregivers play a key role in the examination process of elderly patients, especially with regard to female patients [28]; patients with a low educational level [28, 42]; patients suffering from multiple pathologies [37], Alzheimer’s disease [45], or psychiatric disorders [28]; and patients with bad health conditions or poor self-perception of their health status [24, 28].

Family members and caregivers usually have an encouraging attitude during the medical examination [37, 42], thus improving the communication between the doctor and the patient. The accompanying person’s help can be direct (by asking questions and/or providing the physician with information) or indirect (by encouraging the patient to talk) or by taking notes and explaining the doctor’s indications to the patient. In addition, family members and caregivers provide the patient with moral support, physical assistance, and logistic and organizational help [24, 48, 49].

#### Appropriateness of home visit

According to the only available review [31], the first medico-legal evaluation of an elderly patient should take place at the patient’s home in case of serious mobility impairment resulting in the patient’s inability to reach the doctor’s surgery; high risk of falls, requiring an environmental assessment; serious behavioural problems; end-stage terminal illness; no access to transportation; and refusal to come in for medical examination. In addition, a home visit might be recommended for patients with urgent concerns, or to complete the evaluation of patients already examined in doctor’s surgery or inpatient setting, or as part of a multidimensional assessment programme.

Questions and Jury Panel statements/recommendations are reported in Table 4.

**Table 4:** Medico-legal examination procedures: questions and Jury Panel statements/recommendations

Level of evidence	Questions and Jury Panel statements/recommendations
Experts’ opinions	3.1 Which criteria should be used to establish the minimum time interval before the injured person undergoes medico-legal examination? *Based on the currently available evidence, it is not possible to define a minimum time interval that should be respected before the injured party undergoes medico-legal examination*.
Observational studies (4 of high quality, 3 of average quality), and the experts’ opinions	3.2 Should the time required to examine an older person differ from that required for other age ranges? *The examination time required for an elderly patient is different from that required for patients of other age ranges.* 3.3 Which is the appropriate minimal duration of a medico-legal examination of an older person? *Based on the currently available evidence, it is not possible to define a minimal duration for the medico*-*legal examination of an older person. However*, *due to the overall social*, *clinical*, *and managemental complexity*, *a longer duration of the examination compared to a younger patient should be considered. The high heterogeneity of the elderly population and injury types does not allow for a minimal standard duration for the medico*-*legal examination to be established*.
Two systematic reviews of low quality, observational studies (3 of high quality, 2 of average quality, 1 of low quality), and the experts’ opinions	3.4 Under which circumstances should a family member or caregiver attend the medico-legal visit? *The presence of a family member and/or a caregiver during a medico*-*legal visit can be useful in the case of elderly patients with psychophysical disabilities*. *The presence of an accompanying person must comply with the legislation regarding informed consent*. 3.5 How should a family member or caregiver take part in the medico-legal visit? *The presence of a family member or a caregiver can be useful when the accompanying person adopts an encouraging behaviour towards the patient during a medico*-*legal visit*; *in point of fact*, *the accompanying person can improve patient-doctor communication and provide logistic support to patients with physical disabilities*.^a^
A narrative review of very low quality, and the experts’ opinions	3.6 Under which circumstances should the medico-legal visit be made at the injured person’s home? *It is appropriate to consider holding the medico*-*legal visit at the injured patient*’*s home for patients suffering from serious mobility or psychic disability*, *or for those at high risk of falls* (*also for an environmental assessment*), *or suffering from terminal illnesses*, *or with no access to transportation*.^b^

^a^During the public discussion, the conference audience highlighted the importance of communicating the presence of an accompanying family member and/or caregiver to the parties, ahead of the medico-legal visit

^b^During the public discussion, the conference audience raised an issue about who might/should decide whether a home visit is required or not. Following this discussion, everybody agreed that the medico-legal examiner is the only person who can decide on home visits to the injured party

### Multidimensional assessment and scales

#### When an MDA is necessary

The multidimensional assessment (MDA) (often called comprehensive geriatric assessment or CGA) can identify factors influencing the health and functional status of the older person even when the patient has poor self-perceived health conditions [44]. Although well-defined criteria to establish which patients may actually benefit from an MDA are still lacking, the general criteria to consider are the age and the presence of comorbidities or geriatric syndromes [21].

Multidimensional assessments have long been used for the epidemiological study of disability [76, 77] and for the acknowledgement of the handicap status according to the Italian Law 104/1992 [78]. A single observational study shows that the Barthel index is an independent predictor for the acknowledgment of the accompaniment allowance indemnity [38]. In the medico-legal practice, the rating of impairment severity according to severity levels (as provided, for instance, by the American Medical Association’s Guides to the Evaluation of Permanent Impairment [79]), based on MDA scales, should be compulsory especially when primary biological functions are impaired [80], or in case of serious injuries that have consequences on the entire person [81]. However, neither studies on the use of MDA in personal damage assessment in elderly people, nor evidence on assessment domains and tools to be used are available.

#### Assessment domains and tools

Independently of the specific purpose of an MDA, there is international consensus on the minimum set of domains that should be assessed [17], and on the tools to be used [8, 13, 15–21, 38, 44]:

- Clinical status (comorbidity): Cumulative Illness Rating Scale; Charlson Index
- Functional status: Basic Activities of Daily Living-BADL (Katz Index, Barthel Index) and Instrumental Activities of Daily Living-IADL (Lawton-Brody scale)
- Cognitive status: Short Portable Mental Status Questionnaire, Mini Mental Status Examination, Clock Drawing test
- Affective status: Geriatric Depression Scale (15- or 5-item version)
- Social interactions: Oslo-3 Social Support Scale

Some authors suggest using a smaller set of domains (functional, cognitive, and social status) [12].

When specific alterations are found, a II level assessment should be performed (e.g., II level neuropsychological tests).

In some cases, a functional assessment with physical performance measurements, which better define the functional status, may be useful [44]; the most frequently used tests, which can easily be carried out in outpatient settings, are:

- Four-Meter Gait Speed test [20]
- Get-Up and Go test [17, 20]
- Short Physical Performance Battery [8, 13, 14]
- Grip Strength [19]

In literature, several frailty assessment methods are reported; the “physical frailty” and the “cumulative deficit frailty” are the most cited conceptual frameworks [82]. The frailty domain includes the level of functional reserve and the general homeostatic capacity; therefore, this domain can be useful to estimate the overall health status in the older person [13].

The abovementioned tools have been routinely used for decades in geriatric practice. They provide a quantitative assessment of the patient status resulting in a discrete score that allows an accurate comparison between different examinations, when available.

Additional tools for the evaluation of older patients that are validated in Italian are available [32, 36, 40, 41, 46], but evidence on their usefulness in the medico-legal practice of damage assessment is lacking.

Questions and Jury Panel statements/recommendations are reported in Table 5.

**Table 5:** Multidimensional assessment (MDA) and scales: questions and Jury Panel statements/recommendations

Level of evidence	Questions and Jury Panel statements/recommendations
One observational study of high quality, theoretical/doctrinal documents, synthesis of evidence documents, and the experts’ opinions	4.1. When should a medico-legal expert obtain or perform a multidimensional assessment (MDA) of the older person in order to assess personal damage? *It is recommended to perform an MDA depending on the complexity of the case*, *namely when high comorbidity or significant alterations of the motor or cognitive functional status are present*, *especially in people aged over 75*.
Two observational studies of high quality, guidelines (1 of high quality, 1 of average quality), synthesis of evidence documents, and the experts’ opinions	4.2 Which domains should always be assessed? *In the medico*-*legal practice*, *the MDA of an older patient with a significant reduction or a loss of autonomy, and in relation to the complexity of the case*, *should include the evaluation of the following domains: clinical status* (*presence of comorbidity*); *functional status* (*BADL*, *IADL*); *cognitive status*; *psycho*-*affective status*; *social interactions*, *and environmental context.* 4.3 Which are the recommended assessment tools? *Several tools that can be used to perform MDAs of older patients have been available for a long time in clinical practice. Nevertheless*, *no formally validated multidimensional tools are available in the personal damage assessment context*. *To properly perform a medico*-*legal assessment of personal damage*, *MDA tools should be the ones used for the pre-injury status assessment*. 4.4 Which assessment tools of older people’s functions validated in Italian are most useful for medico-legal purposes? *The tools generally used in clinical practice for first-level screening can also be used for medico*-*legal purposes*. *In selected cases*, *II level assessment screening might be required*. *This is normally performed by a specialist in the field* (*geriatrician*, *psychiatrist*, etc.) *and the most appropriate tools are chosen according to the evaluation purposes*s

### Limitations and areas requiring further investigation

Most of the available studies on traumatic injury outcomes consider the older person as an *unicum*, without taking into account the heterogeneity of the elderly population with regard to several critical factors: global health conditions, frailty, odds of recovery, and social relationships and social capital. Even the most used assessment tools are generally not adequate for the older population. Further studies that consider these factors and investigate the relations between injury outcomes and pre-injury conditions are required. Also, additional studies are needed to validate, in the medico-legal context, the evaluation tools used to assess functioning of older people and the usefulness, for the purposes of personal damage assessment, of the frailty scales and the bio-psycho-social model issued by the World Health Organization [77]. The latter allows for a detailed evaluation of negative outcomes of biological phenomena. As of today, the available evidence does not allow us to identify qualitative indexes of the medico-legal activity other than considering longer examination duration when the injury victim is an older person.

Finally, some members of the Jury Panel required an in-depth discussion of the most appropriate criteria for the monetary quantification and settlement of personal damage in older people, considering, in addition, the possible settlement by an annuity. On the contrary, other Jury Panel members expressed their disagreement on the inclusion of compensation settlement issues, or, in any case, for the mention of possible annuity compensation, in the present report.

The consensus conference referred to personal damage assessment, with a focus on third-party liability. Nevertheless, for the medico-legal discipline unity, the methodological recommendations issued during the Conference may be useful to other contexts of damage assessment, or to other medico-legal evaluation fields.

## Data Availability

Data and material are available from the corresponding author upon reasonable request (francesca.ingravallo@unibo.it).

## Appendix 1. Consensus conference organogram

### Promoters

Società Italiana di Medicina Legale e delle Assicurazioni (SIMLA) – Italian Society of Legal and Insurance Medicine.

Federazione delle Associazioni dei Medici Legali Italiani (FAMLI) – Federation of the Italian Associations of Medico-Legal Experts.

Consulta Nazionale dei Giovani Medici Legali Universitari – National Board of Young Medico-legal Experts.

Centro di studio e ricerca sull’invecchiamento - Ageing Research Centre, Department of Medical and Surgical Sciences (DIMEC), University of Bologna.

Consensus conference was held under the patronage of Società Italiana di Gerontologia e Geriatria (SIGG) - Italian Society of Gerontology and Geriatrics.

### Promoting committee

Gloria Luigia Castellani, Medico-legal expert, FAMLI delegate, Verona.

Alessandro Feola, Medico-legal expert, National Board of Young Medico-legal Experts delegate, University of Campania “Luigi Vanvitelli”.

Paola Forti, Geriatrician, Assistant Professor, DIMEC Ageing Research Centre delegate, University of Bologna.

Francesca Ingravallo, Medico-legal expert, Associate Professor, DIMEC Ageing Research Centre delegate, University of Bologna.

Andrea Molinelli, Medico-legal expert, Associate Professor, SIMLA delegate, University of Genoa.

### Scientific technical committee

Gloria Luigia Castellani, Medico-legal expert, FAMLI delegate, Verona.

Alessandro Feola, Medico-legal expert, National Board of Young Medico-legal Experts delegate, University of Campania “Luigi Vanvitelli”.

Paola Forti, Geriatrician, Assistant Professor, DIMEC Ageing Research Centre delegate, University of Bologna.

Francesca Ingravallo, Medico-legal expert, Associate Professor, DIMEC Ageing Research Centre delegate, University of Bologna (Coordinator).

Maria Lia Lunardelli, Geriatrician, SIGG delegate, University Hospital Policlinico S.Orsola Malpighi, Bologna.

Andrea Molinelli, Medico-legal expert, Associate Professor, SIMLA delegate, University of Genoa.

Luca Vignatelli, Neurologist and Methodologist, IRCCS Istituto delle Scienze Neurologiche di Bologna, Local Health Trust of Bologna.

### Scientific secretariat

Matteo Bolcato, Medico-legal expert, Padua.

Ilaria Cerquetti, Medico-legal expert, Fermo.

Andrea De Nicolò, Medico-legal expert, Turin.

Francesca Frigiolini, Specializing Physician, University of Genoa.

Margherita Portas, Medico-legal expert, Verona.

Maria Camerlingo, Literature review expert, Bologna.

### Conference speakers

Graziamaria Corbi, Geriatrician, Assistant Professor at University of Molise, Campobasso.

Ranieri Domenici, Medico-legal expert, former Professor at University of Pisa.

Alessandra Marengoni, Geriatrician, Associate Professor at University of Brescia.

Patrizia Mecocci, Geriatrician, Professor at University of Perugia.

Lorenzo Polo, Medico-legal expert, Pavia.

Carlo Scorretti, Medico-legal expert, former Professor at University of Trieste.

Stefano Volpato, Geriatrician, Professor at University of Ferrara.

Riccardo Zoja, Medico-legal expert, Professor at University of Milan.

### Jury panel

**Panel chair**

Paola Di Giulio, Nurse, Associate Professor at University of Turin.

**Panel members**

Sandra Albertini, Senior Italia delegate, Bologna.

Domenico De Leo, Medico-legal expert, President of the Italian College of Professors of Legal Medicine, Professor at University of Verona.

Francesco De Stefano, Medico-legal expert, SIMLA delegate, Professor at University of Genoa.

Alessandro Dell’Erba, Medico-legal expert, FAMLI President, Professor at University of Bari.

Piergiorgio Fedeli, Medico-legal expert, President of Italian research group on personal injury (GISDAP), Associate Professor at University of Camerino.

Nicola Ferrara, Geriatrician, SIGG delegate, Professor at Federico II University of Naples.

Pasquale Gianniti, Magistrate, Italian Supreme Court delegate, Rome.

Enrico Gili, National Association of Insurance Companies (ANIA) delegate, Rome.

Primiano Iannone, Internist and Gastroenterologist, Head of the National Center for Clinical Excellence, Quality and Safety of Care (CNEC), Rome.

Alessandro Lovato, Lawyer, The Surveillance and Cooperation Body on Civil Justice, Bologna.

Franco Marozzi, Medico-legal expert, FAMLI delegate, Milan.

Massimo Martelloni, Medico-legal expert, President of Scientific Society of Forensic Medicine of Italian National Health Service Hospitals (COMLAS), Lucca.

Massimo Piccioni, Medico-legal expert, National Institute of Social Insurance (INPS) delegate, Rome*.

Patrizio Rossi, Medico-legal expert, National Institute for Insurance against Accidents at Work (INAIL) delegate, Rome.

Marco Trabucchi, Psychiatrist, Italian Association of Psychogeriatrics (AIP) delegate, University of Tor Vergata, Rome.

*Dr. Piccioni approved the preliminary consensus document but did not participate in the following steps due to his retirement from INPS.

The Consensus Conference was endorsed by: Italian Ministry of Health; Bar Council of Bologna; Italian Association of Psychogeriatrics (AIP); National Association of Insurance Companies (ANIA); Italian National Institute of Health (ISS); Scientific Society of Forensic Medicine of Italian National Health Service Hospitals (COMLAS); The Surveillance and Cooperation Body on Civil Justice.
